# The endocannabinoid system and ophthalmic pathologies: a review of molecular mechanisms and its implications for clinical practice

**DOI:** 10.3389/fmed.2025.1500179

**Published:** 2025-02-05

**Authors:** Tomasz Charytoniuk, Stanisław Półjanowski, Mateusz Michalak, Karolina Kaźmierczak, Bartłomiej Kałużny

**Affiliations:** Department of Ophthalmology, Collegium Medicum, Nicolaus Copernicus University, Bydgoszcz, Poland

**Keywords:** ECS, endocannabinoid system, retina, glaucoma, diabetic retinopathy, CB receptor

## Abstract

Within the last decade the role of the endocannabinoid system (ECS) has been a significant part of ophthalmic research, including both ocular physiology and the development of eye pathologies. It is known that this widespread cell-signaling system is involved in retinal neurobiological processes, including visual signal processing, as well as neurotransmission. Furthermore, various research indicated the involvement of ECS in the molecular basis of various pathologies, mostly glaucoma, diabetic retinopathy, and age-related macular degeneration (AMD). Therefore, the researchers believe that this biological system, its receptors, pathways, and ligands might be considered as an auxiliary compound to reduce the number of patients suffering from ophthalmic diseases. Despite presented in the literature effects of the endocannabinoid system in the eye, none of the current ECS reviews presented a comprehensive description of the endocannabinoid system, its compounds, and, subsequently ophthalmic disorders. Thus, the aim of this review was to summarize all the major data, including the most up-to-date research, concerning a correlation between the endocannabinoid system and the major ophthalmic pathologies.

## 1 Introduction

The endocannabinoid system (ECS) is considered to be one of the most widely studied biological systems within the last decade (1). In recent years, it was demonstrated that this widespread cell-signaling system is a crucial regulator of energy homeostasis and is broadly involved in a multitude of physiological processes playing an essential modulatory role in different tissues, including the eye (2). Moreover, the ECS was associated with the molecular basis of various pathologies. Although majority of studies indicating a correlation between the endocannabinoid system and health disorders deliberated on metabolic and neurodegenerative disorders, e.g., type 2 diabetes (T2DM) or Alzheimer’s disease, researchers demonstrated a pivotal role of the ECS in the eye (3, 4). The role of this biological system in ocular physiology and the development of eye pathologies has been a significant part of ophthalmic research within the last decade, including studies concerning glaucoma or diabetic retinopathy (DR) (5, 6). The endocannabinoid pathway includes elementarily G-protein-coupled receptors, known as cannabinoid receptors CB1 and CB2, and the endogenous agonists of these receptors, known as endocannabinoids—principally anandamide (AEA, N-arachidonoylethanolamine) and 2-arachidonoylglycerol (2-AG) (7). It is known that both CB1 and CB2 receptors are diffusely distributed throughout the organism, i.e., in the gastrointestinal tract, and cardiovascular system, as well as in the eye and central nervous system (8). Considering the endocannabinoids, those molecules are synthesized from omega-3 (docosahexaenoic acid, DHA and eicosapentaenoic acid, EPA) or omega-6 (arachidonic acid, AA) long-chain polyunsaturated fatty acids (LC-PUFAs). Although endocannabinoids are mediators able to bind classic cannabinoid receptors, the non-cannabinoid ECS receptors, e.g., transient receptor potential vanilloid type 1 (TRPV1), G protein-coupled receptor 55 (GPR55), and peroxisome proliferator-activated receptors *α* and *γ* (PPARα and PPARγ), present in the eye, might also be widely activated via these lipid molecules. While endocannabinoids constitute a large group of cannabinoids, we may also distinguish two other classes—synthetic cannabinoids, as well as phytocannabinoids, e.g., cannabidiol (CBD) or delta-9-tetrahydrocannabinol (Δ9-THC), that were also identified as the essential modulators of the endocannabinoid system, also in the eye (9, 10).

Eye disease epidemiology is a very alarming issue since the number of individuals suffering from eye disorders, including glaucoma, age-related macular degeneration (AMD) or DR is high and growing among the global population. Therefore, novel clinical therapies, including the use of the endocannabinoid system, are being widely sought. Despite presented in the literature effects of the endocannabinoid system in the eye, none of the current ECS reviews presented a comprehensive description of the endocannabinoid system, its receptors, and subsequently major ophthalmic disorders linked. Thus, this review significantly contributes to this area of research as it summarizes current data concerning the endocannabinoid system in terms of major ophthalmic pathologies.

## 2 Glaucoma and the endocannabinoid system

Glaucoma is a group of optic neuropathies characterized by degeneration of retinal ganglion cells (RGCs) and their axons, resulting in a visual field loss (11). It is commonly known that glaucoma is one of the leading causes of irreversible blindness worldwide (12). Glaucomatous disease-related vision loss has not only disastrous consequences for patients’ quality of life but also imposes high economic costs, especially in the late stages of the disease (13). Thus, early diagnosis and adequate treatment play a pivotal role in preventing future disability. One of the major risk factors for glaucoma is intraocular pressure (IOP), and its reduction is the only proven modifiable method to treat this disease (14). Currently, most of the glaucoma therapies target IOP (15, 16). However, glaucoma progression might be observed despite IOP within normal limits, which involves mechanisms beyond increased IOP (17, 18). Consequently, novel neuroprotective methods for glaucoma prevention and treatment are still being sought, and a major focus is placed on the modulation of intraocular pressure and neuroprotection (19).

### 2.1 Modulation of IOP and the endocannabinoid system

IOP hinges upon the equilibrium between the production and outflow of aqueous humor. Synthesized in the ciliary body, aqueous humor is subsequently transferred into the posterior chamber via various transporters localized on the bilayered ciliary epithelium. The outflow of aqueous humor from the eye involves two conduits: through the trabecular meshwork, Schlemm’s canal to the episcleral veins, called the conventional pathway and the uveoscleral (unconventional) pathway, where aqueous humor flows through the iridocorneal angle, then the ciliary body and exits through the supraciliary and suprachoroidal spaces (20).

In the 1970s, Hepler et al. observed a reduction in intraocular pressure in healthy young adults after smoking marihuana and this study was pioneering research in terms of the correlation between ECS and the eye (21). Since then, research has been ongoing to understand the components and mechanics of the ocular endocannabinoid system and its use as a novel, potential antiglaucoma agent target. Numerous studies revealed that the IOP-decreasing effect of the cannabinoids might be associated with a reduction of aqueous humor production as well as an increase of trabecular and uveoscleral outflow (22, 23). In addition, a few studies, including one by Chen et al. reported low concentrations of ECS ligands: 2-AG and palmitoylethanolamide (PEA) in the eyes of patients with glaucoma (6, 24). Among two ECS receptors, according to Laine et al., the CB2 receptor does not mediate IOP-lowering properties (25). Therefore, the CB1 receptor appears to be the major contributor to reducing the IOP (26, 27). In support of this, Szcześniak et al. demonstrated in an animal model that the IOP-lowering effect of a synthetic cannabinoid—WIN 55,212-2 was not abolished by the CB2 receptor antagonist AM630, while the CB1 receptor-blocking cannabinoid AM251 reduced this effect (28). Statistically significant IOP lowering effect of CB1 receptor agonists was documented in several human studies, including WIN 55,212-2 and phytocannabinoid Δ9-tetrahydrocannabinol (Δ9-THC) (10, 29). WIN 55,212-2 applied topically, decreases the intraocular pressure by 20 to 31% depending on the dose in patients with glaucoma resistant to standard treatment (10). Interestingly, some studies indicated that sublingual administration of *Δ*-9-THC significantly reduced the IOP compared to placebo (23.5 vs. 27.3 mm Hg) (29). However, CB1 receptor agonists may also mediate IOP decrease via receptor targets distinct from CB1 (28, 30, 31). For example, G protein-coupled receptor 18 (GPR18) is a cannabinoid-related receptor proved to be expressed in the ciliary epithelium, cornea, and the trabecular meshwork of the murine eye. GPR18 agonists, such as Abn-CBD and NAGly (a metabolite of AEA), decrease IOP independently of CB1 or CB2 receptors in the animal model (30). However, no study confirmed GPR18 presence in the human eye yet. Moreover, some studies indicated that GPR55 activation might increase outflow of aqueous humor and lower IOP (32). Interestingly, Keppel Hesselink et al. reported that PEA, a ligand for GPR55, supplemented orally for 2 months in human showed a significant IOP lowering effect, surprisingly with excellent tolerability, and presented this molecule as a potent glaucoma treatment factor (33). Beyond this, 𝛽-adrenergic receptors (*β*ARs) are, at least to some extent, responsible for the IOP-lowering effect of CB1 receptor activation in mice (31). Catecholamines, such as isoproterenol and norepinephrine, are *β*AR agonists and are known to facilitate aqueous humor outflow by trabecular and uveoscleral pathways, which decreases IOP and slows down glaucoma progression. On the other hand, *β*AR blockage is also proved to reduce IOP by decreasing aqueous humor production. Interestingly, cannabinoid agonists, *β*AR antagonists, and *β*AR agonists have not decreased IOP in genetically lacking 𝛽-adrenergic receptors (*β*AR(−/−)) or the CB1 receptor (CB1(−/−)) wild-type mice. In addition, desensitization of the *β*ARs and related to this reduction of catecholamines, abolished the IOP lowering properties of cannabinoid agonist WIN 55,212-2 in mice (31). These findings strongly suggest a role for cannabinoids as indirect ocular sympatholytic agents, which reduce IOP despite lowering norepinephrine secretion.

Another interesting mechanism of ocular hypotensive properties of cannabinoids is facilitating cyclooxygenase-2 (COX-2) expression and consequently increasing the synthesis of matrix metalloproteinases and prostaglandin derivatives. Rösch et al. proved that cannabinoid agonists AEA and Δ9-THC enhanced the production of prostaglandin E2 (PGE2) and metalloproteinases 1, 3, and 9 (34). Since AEA and 2-AG can be converted in reactions catalyzed by COX-2 to prostaglandins and chemically related prostamides, whose analog, e.g., bimatoprost, plays a crucial role in glaucoma treatment, it is believed that endocannabinoids can modulate the uveoscleral outflow, resulting in IOP reduction (35, 36). Interestingly, specific loss of COX-2 expression appears to be linked to the occurrence of primary open-angle glaucoma, as COX-1 and COX-2 are localized in the epithelium of the ciliary body in healthy human eyes (37). However, it should be noted that the exact mechanism of the above actions still needs to be thoroughly explained. Table 1 provides a summary of significant research examining the effect of the endocannabinoid system on intraocular pressure.

**Table 1:** Summary of major studies presenting the effect of the endocannabinoid system on intraocular pressure.

Authors and year of publication	Study subjects	Substance/molecule with dose	ECS mechanism	Main outcomes
Hepler et al. 1971 (21)	11 healthy subjects	**Δ9-THC** Inhalation of dried marihuana 2 grams of 0.9%	CB1 receptor agonism	Approx. 25% IOP ↓ (marihuana decreases IOP in healthy individuals)
Pate et al. 1995 (90)	Treated, normotensive eyes of albino rabbits	**AEA** Topical ocular administration 25 μL of 62.5 μg	CB1 agonism	4.4 ± 1.7 mmHg IOP ↓. Initial IOP ↑ may occur
	Untreated eyes of pigmented rabbits			2.1 ± 0.3 mmHg IOP ↓. No initial IOP ↑ was observed
Porcella et al. 2001 (10)	8 patients with glaucoma resistant to conventional therapies	**WIN 55212-2** Topical ocular administration of two drops 50 μL of 25 or 50 μg	CB1 receptor agonism	20 and 31% IOP ↓ with the two dosages used 25 μg and 50 μg The IOP-lowering effects of WIN55212-2 were time and dose dependent in glaucomatous patients
Tomida et al. 2006 (29)	6 male patients with ocular hypertension or early primary open angle glaucoma	**Δ9-THC** Sublingual administration 5 mg	CB1 receptor agonism	IOP ↓ (23.5 mmHg) versus placebo (27.37 mmHg, *p* = 0.026)
		**CBD** Sublingual administration 20 mg and 40 mg	CB1 receptor antagonism?	Sublingual administration of 20 mg CBD did not ↓ IOP, whereas 40 mg CBD produced a transient IOP ↑
Gagliano et al. 2011 (91)	42 patients with raised IOP, treated insufficiently with timolol 0.5%	**PEA** Oral administration 300 mg	Competing substrate with AEA for the FAAH active site	3.2–3.5 ± 1.2–1.3 mmHg IOP ↓ starting with 19–24 mmHg

ECS, endocannabinoid system; Δ9-THC, delta-9-tetrahydrocannabinol; IOP, intraocular pressure; CBD, cannabidiol; AEA, anandamide; PEA, palmitoylethanolamide; FAAH, fatty-acid amide hydrolase 1; CB1 receptor, cannabinoid receptor type 1; CB2 receptor, cannabinoid receptor type 2.

### 2.2 Neuroprotection

The neuroprotective effect of endocannabinoids and modulators of their metabolism is the second essential pillar of endocannabinoid system regulation in glaucoma treatment. A key concern in the pathophysiology of glaucoma is increased apoptosis of RGCs, which, in addition to elevated intraocular pressure and being partially associated with it, is the primary cause of the disease’s progression. An elevated IOP is the reason for the optic disc compression and subsequent irreversible reduction of peripapillary retinal nerve fiber layer (RNFL) thickness. Knowledge of the neuroprotective properties of the ECS and the CB2 receptor, in particular, has increased in recent years. It was demonstrated that the CB2 receptor presents anti-inflammatory and anti-apoptotic effects, which may represent a promising therapeutic target for neuroprotection in patients suffering from glaucoma (38, 39). Studies evaluating cannabinoids’ neuroprotective effects in human models remain scarce. However, numerous animal studies illustrate enhanced outcomes for RGCs survival. A 75% decline in RGC loss was shown after the administration of Δ9-THC for 20 weeks in rodent ocular hypertension model with adult male albino Sprague–Dawley rats, possibly through a reduction in IOP (40). A similar outcome was observed in a Wistar rat model of mechanical optic nerve injury unrelated to IOP. In this case, the administration of URB 597 that is an inhibitor of fatty acid amide hydrolase (FAAH), a primary metabolic enzyme for AEA, improved the ganglion cells survival by significant increase of AEA with a simultaneous decrease in its metabolites. However, the neuroprotective effect was observed only in younger subjects (41).

Cannabinoids’ neuroprotective potential may also be due to the abolishment of peripapillary circulation abnormalities. Interestingly, endogenous cannabinoids such as AEA can, in human models, decrease endothelin-1 levels, which are elevated in glaucoma patients, potentially playing a neuroprotective role by increasing blood supply to the optic nerve head (42–44). Since optic disc hypoperfusion is an identified factor of glaucomatous disease progression, the anti-inflammatory effects of cannabinoids significantly contribute to their neuroprotective profile by mitigating nitric oxide and inflammatory cytokine production (45). Moreover, cannabinoids cause glutamate release inhibition, which may play a crucial role in glaucoma progression (46), Inhibition of nitric oxide and glutamate release alleviates oxidative stress and prevents RGC loss, among others *via* excitotoxicity (41, 47). Furthermore, decreased glutamate results in the activation of pre-synaptic CB receptors, facilitating neuronal excitability and synaptic plasticity. Interestingly, studies on mice model demonstrated that the endocannabinoid compounds exhibit a neuroprotective activity via attenuation of striatal degeneration and microgliosis, improvement of motor deficits, as well as its effect on NFκB-inhibitory proteins and down-regulation of proinflammatory markers (48, 49). Table 2 summarizes key research that analyzed the correlation between the endocannabinoid system and neuroprotection.

**Table 2:** Summary of major studies presenting the neuroprotective effect of the endocannabinoid system in the eye.

Authors and year of publication	Study subjects	Substance/molecule with dose	ECS mechanism	Main outcomes
Ronco et al. 2006 (44)	Cultured human umbilical vein endothelial cells	**AEA** > 5 μmoL/L	AEA inhibits endothelin-1 production	Potential ↑ of peripapillary blood supply
Crandall et al. 2007 (40)	Albino Sprague–Dawley rats with an ocular hypertension model	**Δ9-THC** Intraperitoneal injection 5 mg/kg	CB1 receptor agonism	Δ9-THC treatment effective in reducing RGC loss (possibly *via* a IOP ↓).
Slusar et al. 2013 (92)	Fischer-344 rats post optic nerve axotomy	**URB597** Intraperitoneal injection 0.3 mg/kg	CB1 receptor agonism	Improvement of the ganglion cells survival only in younger subjects
Leonelli et al. 2013 (47)	Wistar rats	**Capsaicin** Intravitreal injections 100 μM	TRPV1 agonism	Potential role for TRPV1 channels in physiopathological retinal processes mediated by NO, which at least in part involve glutamate release

ECS, endocannabinoid system; IOP, intraocular pressure; AEA, anandamide; Δ9-THC, delta-9-tetrahydrocannabinol; TRPV1, transient receptor potential vanilloid type 1; URB597, cyclohexylcarbamic acid 3′-carbamoyl-biphenyl-3-yl ester; CB1 receptor, cannabinoid receptor type 1; CB2 receptor, cannabinoid receptor type 2.

### 2.3 A new model of glaucoma therapy

The ECS was firstly taken under consideration as a new potential therapeutic strategy against glaucoma in the 1970s (21). For a few decades, scientific attention was focused on the IOP-lowering properties of ECS, primarily *via* activation of the CB1 receptor. However, no cannabinoid drugs have been approved for glaucoma treatment yet. So far, critical issues such as systemic side effects, including psychotropic effect, short-acting time, and tachyphylaxis attest against cannabinoids usage as anti-glaucoma drugs (44, 50, 51). Nevertheless, among all forms of cannabinoid application, the oral one appears the most promising. Recent studies suggest focusing on a better understanding of CB2 receptor activation, which can be beneficial for glaucoma patients because of its neuroprotective effect, which consists of anti-oxidative, anti-inflammatory, and vasodilatory properties. Furthermore, activation of CB2 receptors is not associated with the psychotropic effects. We believe that clinical trials on larger populations and extended follow-up times are needed to clarify the molecular mechanisms and long-term effects of cannabinoids in glaucoma treatment.

## 3 Uveitis and the endocannabinoid system

Uveitis might be defined as an inflammatory process of the particular parts of the uveal tract: the iris, ciliary body, and choroid. This might also be described anatomically, based on the affected element, as anterior, intermediate, posterior, or throughout the eye, named panuveitis. Although most uveitis cases are idiopathic, others such as trauma, infection, and systemic diseases, e.g., autoimmune, are also identifiable causes (52). Toguri et al. were the first to demonstrate the potent role of topical cannabinoid treatment as an anti-inflammatory agent for uveitis. In this study, topical application of HU 308, a potent and selective CB2 receptor agonist, significantly reduced inflammatory mediator release and leukocyte-endothelial adhesion in a model of endotoxin-induced uveitis by intraocular injection of lipopolysaccharide (LPS) in rats. HU-308, decreased leukocyte-endothelial adhesion and attenuated the release of pro-inflammatory mediators (TNF-*α*, IL-1β, IL-6, INF-*γ*, CCL5 and CXCL2) and the transcription factors (NF-κβ and AP-1), responsible for the transcription of pro-inflammatory genes in anterior uveal tissue obtained from Male Lewis rats (39). Interestingly, this study reported that CB2-activating drugs achieved better results than three examples of current clinical anti-inflammatory treatment of uveitis, i.e., dexamethasone, prednisolone, nepafenac. On the other hand, the application of cannabinoids in experimental uveitis might also present undesired consequences. Altinsoy et al. reported that AEA, a partial CB1 receptor agonist, injected intravitreally in a rabbit model of LPS-induced uveitis increased all measured inflammation factors (53). Subsequently, inflammation decrease by CB1 receptor antagonism with AM251 was noted. On the contrary to the results of the latter study, Toguri et al. discovered that intravenous application of the cannabinoid WIN 55212-2 might lead to an anti-inflammatory effect in experimental uveitis generated by systemic LPS injection in rats, but the beneficial properties of WIN 55212-2 were abolished by CB2 receptor antagonism (54). Interestingly, Porter et al. presented the anti-inflammatory effects of selective CB2 receptor agonists administrated *via* the topical route, which were proved effective in reducing the adhesion of leukocytes to the iris microvasculature in mice model of uveitis induced by endotoxins (55). The data above strongly support the selective targeting of CB2 receptor for the treatment of ocular inflammatory diseases, among them uveitis. Table 3 summarizes key research that analyzed the correlation between the endocannabinoid system and uveitis.

**Table 3:** Summary of major studies presenting the effect of the endocannabinoid system on uveitis.

Authors and year of publication	Study subjects	Substance/molecule with dose	ECS mechanism	Main outcomes
Altinsoy et al. 2011 (53)	Albino male rabbits with LPS- induced uveitis	**AEA** Intravitreal injection 50 μL (10^−5^ M)	CB1 receptor agonism	↑ Uveitis
	Albino male rabbits with LPS- induced uveitis given intravitreal injection of 50 μL AEA (10^−5^ M)	**AM251** Subtenon injection 0.1 mL (10^−5^ M)	CB1 receptor antagonism	AM251 administration reversed some components of AEA induced exacerbation to significant extents
Toguri et al. 2014 (39)	Male Lewis rats with LPS- induced uveitis	**HU-308** Topical administration 5 μL 1.5%	CB2 receptor agonism	↓ Leukocyte-endothelial adhesion ↓ Release of pro-inflammatory mediators
Toguri et al. 2015 (54)	Lewis rats with LPS- induced endotoxemia	**WIN 55212-2** Intravenous administration 1 mg/kg	CB2 receptor agonism	↓ Leukocyte-adhesion ↑ Iridial microvascular blood flow
Porter et al. 2019 (55)	Male BALB/c wild type CB2R knockout mice	**RO6871304** Topical administration 1.5%	CB2 receptor agonism	

LPS, lipopolysaccharide; ECS, endocannabinoid system; IOP, intraocular pressure; AEA, anandamide; CB1 receptor, cannabinoid receptor type 1; CB2 receptor, cannabinoid receptor type 2.

## 4 Retina and the endocannabinoid system

### 4.1 Retinal neurobiology and the ECS

The presence of different ECS components in the eye suggests an essential role of this biological system in ocular neurophysiology. Moreover, the activity of the ECS was described in both retinal neurobiology and pathologies involving this light-sensitive, innermost eye tissue (56). Concerning the neurobiological basis, it is known that ECS and cannabinoid signaling are widely involved in several neurophysiological processes, including visual signal processing, neurotransmission, and neuroplasticity (57, 58). Some studies demonstrating a correlation between the retina and the ECS indisputably contributed to a better understanding of the neurophysiology of the eye. However, the exact role of the endocannabinoids and their receptors in the vertebrate retina is still a vast challenge for scientists. Interestingly, Schwitzer et al. showed delayed responses in ganglion cells and bipolar cells in electroretinography as well as delayed visual signaling in regular cannabis users (59, 60). Increased retinal background noise, which is neural activity during resting, is another noted dysfunction in regular cannabis users (61). An animal study conducted by Miraucourt et al. indicated that CB1 receptor activation with WIN 55,212-2 leads to enhanced visual perception via changes in levels of intracellular chloride in retinal ganglion cells (62). Furthermore, a recent Vielma et al. study indicated that CB1 receptor activation affects OFF bipolar cells’ response by selectively regulating GABAergic feedback inhibition (63). Along with this research, Middleton et al. indicated that both cannabinoid agonist WIN55212-2 and the inverse agonist AM251 significantly affect the sensitivity of ON retinal ganglion cells (linked to ON bipolar cells) to cannabinoids in dark-adapted mice retina (64). Some studies indicated that the endocannabinoid receptors might be widely involved in visual functions. A Bouskila et al. study conducted on vervet monkeys demonstrated that non-cannabinoid receptor GPR55 might play a significant role in mediating scotopic vision. Therefore, it is believed that this may offer a new treatment approach to restore night vision, which is weakened in a multitude of eye pathologies (65). All the research may lead to the conclusion that the ECS plays a pivotal role in processing of visual signals in the retina.

### 4.2 Retinal pathologies and the ECS

Even though the endocannabinoid system widely modulates retinal neurophysiology, it is believed that it might be involved in the molecular basis of various retinal pathologies, including diabetic retinopathy or age-related macular degeneration (66). Buraczynska et al. clinical study on individuals with T2DM found that G1359A polymorphism in the CNR1 gene, which encodes the CB1 receptor, is associated with microvascular complications in T2DM, including DR (67). Furthermore, Matias et al. study conducted on the cadaveric DR and AMD eyes indicated changes in endocannabinoids, i.e., AEA and 2-AG, as well as PEA levels in comparison to normal eye. In eyes with DR, significantly higher levels of AEA were found, whereas the PEA level was lower in the retina. In eyes with AMD, the levels of AEA showed a trend toward an increase in the retina (68).

A multitude of research demonstrated that neuroinflammation and oxidative stress play a pivotal role in the development of various retinal pathologies, e.g., diabetic eye disease, AMD, as well as retinal vein occlusion (69, 70). Moreover, some studies indicated a relevant correlation between the ECS and both neuroinflammation and oxidative stress. El-Remessy et al. study, conducted on human primary retinal endothelial cells (HREC) exposed to high glucose and rodent model of DR, revealed that genetic deletion of CB1 receptor, as well as administration of its antagonist SR141716, significantly inhibit retinal cell apoptosis, attenuate the oxidative stress and diminish inflammatory signaling (71). Furthermore, a recent study conducted by Spyridakos et al. indicated that topical administration of CB1 receptor antagonist, i.e., SR141716 and CB2 receptor agonist, i.e., AM1710 in a rodent model of the early-stage diabetic retinopathy (ESDR) might lead to attenuation of nitrative stress, diabetes-induced apoptotic cell death, vascular leakage, as well as results in protection of RGC axons (72). Recently Grether et al. indicated that CB2 receptor agonism with RG774 decreases retinal vascular permeability and leukocyte adhesion in rodent model (73). Therefore, it is believed that performing a treatment strategy aimed at ECS compounds, e.g., cannabinoid receptors, might be a promising tool in the treatment of diabetes complications, including DR.

Considering the correlation between the ECS and age-related macular degeneration, Wei et al. *in vitro* study on primary human retinal pigment epithelial (RPE) cells, a cellular model of AMD, indicated that inhibition of CB1 receptor downregulates oxidative stress signaling and facilitates PI3K/Akt pathway activation. Therefore, showed a novel treatment strategy for AMD with the ECS component (74). Interestingly, an *in vitro* Hytti et al. research, also on RPE human cells, demonstrated that activation of the CB2 receptor with JWH-133 might lead to the development of retinal inflammation and oxidative stress-induced cell death (75). Knowing the fact that the neuroinflammation in RPE cells and impairment in RPE function significantly contribute to the development of AMD, cannabinoid receptors might be considered as possible molecular targets for the treatment of macular degeneration.

Peroxisome proliferator-activated receptors (PPARs) are nuclear receptors that play a pivotal regulatory role in energy homeostasis and metabolic function in various tissues, including the retina (76, 77). Within the last decade, PPARs and their agonists were indicated to present anti-inflammatory, anti-oxidative, and anti-angiogenic properties that constitute the basis of various ocular diseases (78). A wide range of studies, both animal models and clinical trials, indicated that agonists of PPARs might have a therapeutic potential for the prevention and treatment of DR (79). Moreover, a recent Qui et al. study indicated that fenofibrate acid, a peroxisome proliferator–activated receptor-alpha agonist, reduced choroidal neovascularization (CNV) in mice model of neovascular AMD (80). Interestingly, the endocannabinoid system was described as a significant modulator of PPARs (56, 81). It is known that a large number of both endogenous and exogenous lipid molecules, including phyto- and endocannabinoids, might activate PPARs (81). Recently, Ye et al. indicated both *in vitro* and *in vivo* that PEA might reduce retinal neovascularization and fibrotic changes in proliferative retinopathy (PR) and neovascular AMD *via* PPARα activation (82). Thus, it is believed that PPARs and their broad interaction with the ECS might constitute a promising tool for the treatment of retinal pathologies. In addition to this, it is also worth mentioning that nowadays, plant-derived compounds that may effectively affect metabolism are considered to be very promising agents in terms of future treatment of pathologies associated with inflammatory response and oxidative stress. Most importantly, phytocannabinoids, e.g., CBD, and their anti-inflammatory, anti-oxidative as well as neuroprotective effects were widely demonstrated as a promising treatment strategy for various metabolic pathologies, including DR (83, 84). It was indicated that treatment with cannabidiol may lead to the decrease of oxidative stress, neuroinflammation, and breakdown of the blood-retinal barrier, as well as prevent retinal cell death in diabetic rats (85). Furthermore, Priestley et al. *in vitro* study indicated that fenofibrate acid presents agonism at the CB2 receptor and a partial agonism at the CB1 receptor (86). It is widely known that therapy with fenofibrate may reduce progression of diabetic retinopathy (87) and this can potentially be achieved due to its correlation with the endocannabinoid system and CB receptors.

**Figure 1:**
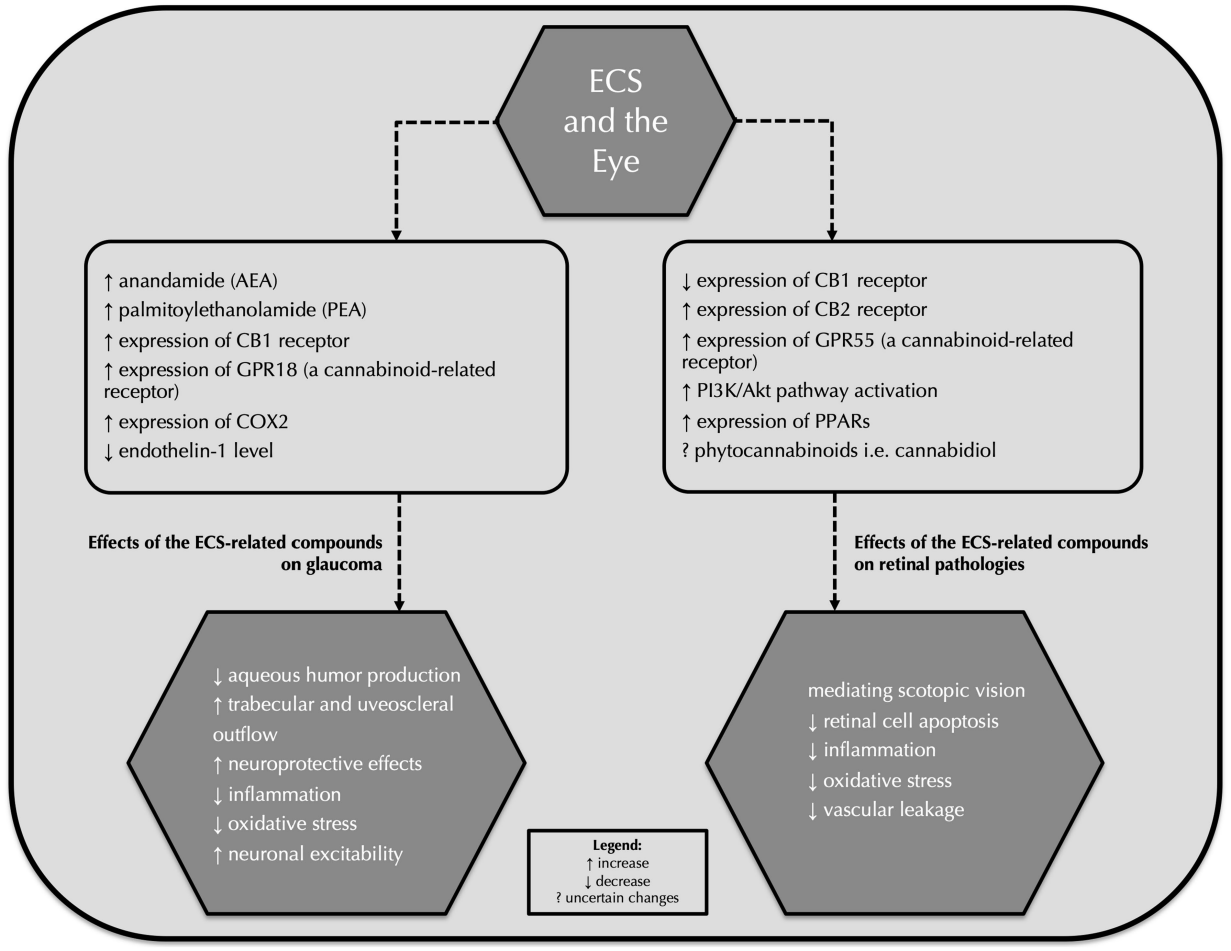
Effects of the endocannabinoid system-related compounds on glaucoma and retinal pathologies. ECS, endocannabinoid system; AEA, anandamide; PEA, palmitoylethanolamide; GPR18, G protein-coupled receptor 18; COX-2, cyclooxygenase-2; PPARs, peroxisome proliferator-activated receptors.

Concerning other retinal pathologies, Imamura et al. indicated both *in vitro* and *in vivo* that the CB1 receptor expression might be upregulated by light exposure and the inhibition of the CB1 receptor with rimonabant, the CB1 receptor antagonist, as well as activation of the CB2 receptor with HU-308, a cannabinoid type 2 receptor agonist, might attenuate light-induced retinal cell apoptosis. Therefore, it is believed that these components of the ECS might constitute a therapeutic approach for light-associated retinal diseases (88, 89). It is reported that enormous progress in the research involving the endocannabinoid system and the development of new therapeutic compounds that interact with cannabinoid and non-cannabinoid ECS receptors might contribute to improving various retinal pathologies. We believe that the endocannabinoid system and its related compounds will constitute a new possible therapeutic approach to target these diseases in the upcoming years despite the fact that there is currently not much research on the subject of ECS and retinal disturbances. Table 4 summarizes key research that analyzed the correlation between the endocannabinoid system and retinal diseases. Figure 1 summarizes effects of the endocannabinoid system-related compounds on glaucoma and retinal pathologies.

**Table 4:** Summary of major studies analyzing correlation between the endocannabinoid system and the retinal pathologies.

Authors and year of publication	Study subjects	Substance/molecule with dose	ECS mechanism	Main outcomes
Wei et al. 2013 (74)	Primary human RPE cells (AMD cellular model)	**SR141716** (no information about dose)	CB1 receptor antagonism	↓ Oxidative stress signaling ↑ PI3K/Akt activation
Hytti et al. 2017 (75)	Primary human RPE cells (AMD cellular model)	**JWH-133** (no information about dose)	CB2 receptor agonism	↑ Retinal inflammation ↑ Oxidative stress-induced cell death
Imamura et al. 2018 (88)	661 W cells (immortalized murine retinal cell line) and male albino ddY mice	**HU-308** 1 to 100 nM (cells) 2 μL of 5 μM (mice)	CB2 receptor agonism	Cells: protective effect on the light-induced death of 661 W cells Mice: ↑ the a- and b-waves of the ERGs, ↑ thickness of the outer nuclear layer of the murine retina after light exposure
El-Remessy et al. 2018 (71)	Human primary retinal endothelial cells (HREC) and C57BL/6J Cb(1)(+/+) and Cb(1)(−/−) mice (rodent model of DR)	**SR141716** Intraperitoneal administration for 11 weeks 10 mg/kg daily	CB1 receptor antagonism	↓ Retinal cell apoptosis ↓ Oxidative stress ↓ Inflammatory signaling
Spyridakos et al. 2023 (72)	Sprague Dawley rats (early stage of DR – ESDR model)	**SR141716**^**1**^ **AM1710**^**2**^ Topical ocular administration for 14 days 10 mg/mL 20 μL of vehicle	^1^ CB1 receptor antagonism ^2^ CB2 receptor agonism	↓ Nitrative stress ↓ Diabetes-induced apoptotic cell death ↓ Vascular leakage Protection of RGCs axons

ECS, endocannabinoid system; DR, diabetic retinopathy; CB1 receptor, cannabinoid receptor type 1; CB2 receptor, cannabinoid receptor type 2; RGCs, retinal ganglion cells; RPE, retinal pigment epithelium; ERG, electroretinogram; ESDR, early stage diabetic retinopathy.

## 5 Conclusion

In conclusion, this review comprehensively summarizes a number of studies, including the most up-to-date research, that concerns a correlation between the endocannabinoid system and the major ophthalmic pathologies. The researchers believe that the ECS, its receptors, pathways, and ligands might be considered as an auxiliary compound to reduce the number of patients suffering from ophthalmic diseases. It is worth noting that the ECS-related prevention and treatment methods might be associated with possible adverse effects. Therefore, further studies are obligatory before implementing novel endocannabinoid system-based methods in clinical practice.
